# Construction and validation of an educational booklet on HIV pre-exposure prophylaxis

**DOI:** 10.1590/0034-7167-2024-0245

**Published:** 2025-09-01

**Authors:** Marina Andreoli Trigo, Karyanna Alves de Alencar Rocha, Marcela Antonini, Daniel de Macêdo Rocha, Henrique Ciabotti Elias, Renata Karina Reis

**Affiliations:** I Universidade de São Paulo. Ribeirão Preto, São Paulo, Brazil

**Keywords:** Educational Technology, Disease Prevention, HIV, Pre-Exposure Prophylaxis, Nursing, Tecnología Educacional, Prevención de Enfermedades, VIH, Profilaxis Preexposición, Enfermería

## Abstract

**Objectives:**

to develop and validate an educational booklet on HIV Pre-Exposure Prophylaxis (PrEP).

**Methods:**

quantitative validation study aimed at developing and validating an educational booklet by experts and the target audience. For validation with experts, we used the Health Education Content Validation Index developed in a virtual environment. For the target audience, we used a questionnaire with questions related to organization, writing style, appearance, and motivation.

**Results:**

thirty-two experts and 13 PrEP users participated in the study. The analysis was structured on the principles of descriptive statistics, and domains that presented a Content Validity Index equal to or greater than 0.80 were considered satisfactory. The final version of the material was made available in online and printed formats.

**Conclusions:**

the educational booklet was developed and validated by experts and the target audience, serving as an educational tool to support self-care and awareness of HIV prevention for PrEP users.

## RESUMEN

**Objetivos:**

elaborar y validar un folleto educativo sobre la Profilaxis Preexposición (PrEP) al VIH**. Métodos:** es un estudio cuantitativo de validación destinado a elaborar y validar un folleto educativo por expertos, con el Índice de validación de Contenido Educativo en salud en ambiente virtual y público-objetivo, mediante un cuestionario sobre organización, estilo de la escritura, apariencia y motivación.

**Resultados:**

participaron del estudio 32 expertos y 13 usuarios de la PrEP. El análisis se estructuró según los principios de la estadística descriptiva, y se consideraron satisfactorios los dominios con un Índice de Validez de Contenido (IVC) igual o superior a 0,80. La versión final del material se puso a disposición en línea y en versión impresa.

**Conclusiones:**

el folleto educativo fue desarrollado y validado por expertos y público-objetivo, y sirve como herramienta educativa para apoyar el autocuidado y concienciar sobre la prevención del VIH entre los usuarios de la PrEP.

## INTRODUCTION

Human immunodeficiency virus (HIV) infection continues to be a global public health challenge, with 85.6 million people infected and 40.4 million AIDS-related deaths recorded since the pandemic began. In 2022, 1.3 million new cases of HIV were diagnosed, resulting in 630,000 AIDS-related deaths^(1)^.

In view of the great concern about controlling the transmission of the virus, during the United Nations summit on Sustainable Development Goals (SDGs) in September 2015, 17 goals were established: among them, goal number 3 is described as health and well-being, which encompasses targets related to the topic. One of its goals is the eradication of AIDS and other neglected diseases. The elimination of AIDS is an important goal for public health, since it claimed approximately 630,000 lives in 2022, according to UNAIDS statistics, demonstrating that even with the advances achieved by science and the joint efforts of nations, aiming to interrupt the chain of transmission and reduce the number of new infections, significant losses still occur^(2)^.

Pre-Exposure Prophylaxis (PrEP) was implemented in Brazil in 2017 for populations exposed to HIV, with indication criteria expanded to the entire population over 15 years old and weighing more than 35 kg, including serodifferent couples and people who use injectable drugs^(3,4)^. PrEP is a daily drug therapy with oral antiretrovirals, safe, with few side effects and with proven protective efficacy of over 90% when used correctly^(4)^. Despite its effectiveness, knowledge about PrEP among the population is still limited or lacking, which hinders its widespread adoption^(5)^. One of the main challenges currently faced by health professionals is how accessible the communication of information about Pre-Exposure Prophylaxis (PrEP) is, meeting the needs and doubts of both individuals and the community in a clear and objective manner^(6)^. The demand for health education is widespread, and there is constant discussion about ways to provide information and scientific updates in an accessible manner to the population, ensuring that they can understand and use content based on reliable evidence. In addition, the way in which this information is made available directly influences the learning process and, consequently, the health of individuals^(7,8)^.

Educational technology is a crucial tool in nursing to promote health prevention strategies, including HIV awareness and prevention methods, such as combination prevention. It is also important for individuals with HIV to maintain their viral load undetectable through treatment, thus interrupting the transmission cycle^(9)^.

Considering the difficulty of the population’s access to clear and objective information about PrEP, as well as the need to strengthen AIDS prevention strategies, as outlined in the third objective of the Sustainable Development Goals (SDGs), it is imperative to create easily accessible and understandable material to promote health education. Thus, this study aimed to create and validate an educational booklet on HIV Pre-Exposure Prophylaxis (PrEP).

## OBJECTIVES

To develop and validate an educational booklet on HIV Pre-Exposure Prophylaxis (PrEP).

## METHODS

### Ethical aspects

The study was approved by the Research Ethics Committee, ensuring compliance with the recommendations of Resolution Number 466/12. It was conducted using the Research Electronic Data Capture Platform (REDCAp)^(10)^ with national coverage.

### Study design, period and setting

This is a quantitative validation study, developed in three stages: construction of the educational booklet, content validation with experts and validation with the target audience, carried out from December 2022 to February 2024. This study follows the guidelines of the Revised Standards for Quality Improvement Reporting Excellence (SQUIRE 2.0)^(11-14)^.

### Construction of the educational booklet

The pre-production stage of the educational booklet consisted of a literature review on HIV Pre-Exposure Prophylaxis, articulated with the recommendations of the Clinical Protocol and Therapeutic Guidelines for Pre-Exposure Prophylaxis (PrEP) (2022) for HIV infection risk and current international scientific evidence, from May to December 2022^(3)^. The publications underwent critical reading in order to extract useful information to be included in the educational booklet, defining the main topics and their objectives.

The production of the educational booklet included layout and infographics to facilitate understanding. A designer specialized in health educational materials was hired for this purpose. The process took place from December 2022 to July 2023, following the guidelines proposed by Moreira (2003) to create effective educational material in terms of language, layout and illustrations.

### Sample and eligibility criteria for validation

To identify and select the experts, a criteria that demonstrate expertise in specific health knowledge on the study topic was necessary, according to criteria adopted by Fehring (1994)^(15)^. In order to validate the educational booklet, we invited PrEP patients undergoing follow-up at the Central Specialty Reference Center of Ribeirão Preto city, between October and December 2023. The inclusion criteria were: being over 18 years old, clinical stability, lucidity and willingness to read the booklet. Data collection was performed in a room in the service to ensure confidentiality. Patients with visual or cognitive impairment were excluded. Participants were selected consecutively during the study period. Free and Informed Consent was obtained from all individuals involved in the study online.

### Expert validation

The validation stage was carried out virtually, reflecting trends in using digital means to facilitate empirical studies, serving as a means for collecting and disseminating results^(16,17)^. Recruitment used the Snowball method, as described by Fernandes and Carvalho (2003)^(18)^, adapted for use in virtual social networks as a means of collecting data and disseminating scientific results.

The evaluation of the educational booklet was conducted through a self-administered and virtual questionnaire, inserted in the REDCap platform^(12)^ and adapted from the Nursing Diagnosis Content Validation model proposed by Fehring (1994)^(15)^. The questionnaire used a Likert-type scale to analyze the booklet in different aspects, from its objectives to its structure, presentation and relevance. Participants were able to provide suggestions and observations on the evaluated attributes, including identifying errors or requesting the addition of relevant content.

### Target audience validation

During PrEP follow-up consultations at the specialized service, one of the researchers from the AIDS and STD Center (NAIDST) approached users and invited them to participate in the validation. After acceptance, participants received a semi-structured questionnaire on sociodemographic data, a validation questionnaire prepared by the researchers, and the educational booklet in virtual format. The validation questionnaire, adapted from a previous study, contained 20 questions grouped into organization, writing, appearance, and motivation. Responses were categorized as totally agree/agree (positive responses) and disagree/totally disagree (negative responses).

### Analysis of results and statistics

To analyze the experts’ responses, the Content Validation Index (CVI)^(17)^ was used, which measures the proportion of agreement among evaluators. Consensus was considered satisfactory when the CVI was equal to or greater than 80%. The CVI allowed verifying the experts’ agreement regarding the representativeness of the content. To evaluate the users’ responses, the Agreement Index (AI)^(17-21)^ was used, which calculates the number of agreements divided by the total number of evaluations. An agreement greater than or equal to 0.8 was considered satisfactory. Sociodemographic data were analyzed descriptively, presented in tables, and contributed to possible modifications to the booklet, resulting in its final version. The study was approved by the Research Ethics Committee of the Ribeirão Preto School of Nursing, University of São Paulo.

## RESULTS

### Construction of the educational booklet

**Figure 1:**
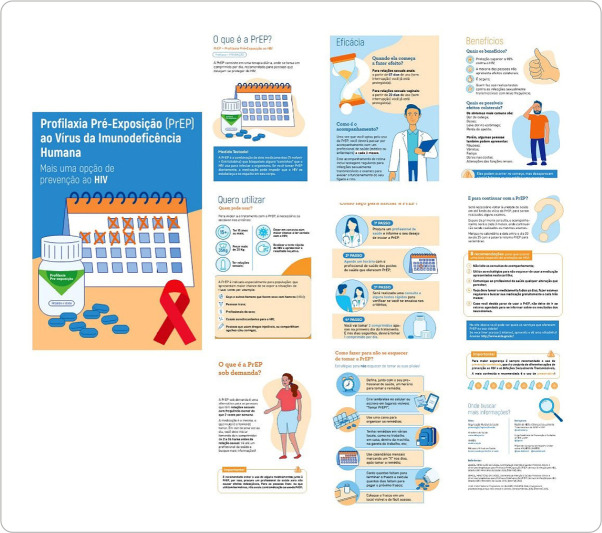
Educational booklet: Pre-Exposure Prophylaxis to the Human Immunodeficiency Virus

*Source: Educational booklet on PrEP, 2024.*

The educational booklet entitled Pre-Exposure Prophylaxis for the Human Immunodeficiency Virus: another option for preventing HIV (2024) has 16 pages, divided into five topics with illustrations and short informative texts. To create the educational booklet, basic information about PrEP^(22)^ was gathered from the Clinical Protocol and Therapeutic Guidelines for Pre-Exposure Prophylaxis (PrEP) for Risk of HIV Infection (2022), and a professional designer was hired, considering the distribution of elements in a cohesive way, using colors associated with the health theme and aesthetically pleasing, as seen in Figure 1.

The first topic of the educational booklet, entitled “Pre-Exposure Prophylaxis for HIV, how does it work?”, describes how the medications work and the indication for PrEP. The second topic, “Efficacy and Benefit”, addresses issues such as when PrEP starts to take effect, the necessary monitoring, benefits and side effects. The third topic, “I want to use it”, answers who can use PrEP, how to start and continue its use, based on the Brazilian protocol. The content was adapted to be accessible, free of complex technical terms and playful, aiming to reach and be understood by the population in different contexts. The fourth topic, “Strategies for not forgetting to take it”, offers six simple guidelines to improve adherence to PrEP, such as note-taking and the use of alarm clocks. Finally, the fifth topic, “Where to find more information”, lists official websites and electronic addresses for additional information about PrEP^(9)^.

Infographics were inserted throughout the text to facilitate reading, and elements were created to reinforce the written message. The predominant color used was blue, which Fernandes and Araújo (2018)^(23)^ describe as a color capable of bringing a sense of calm and relaxation and dispelling the feeling of rushing and agitation that individuals carry every day.

### Validation of the educational booklet by experts

The educational booklet validation stage involved the participation of 32 experts. These were predominantly nursing graduates, 29 (93.5%), and female, 18 (56.3%) from different regions of the country. The average length of experience in the field was 8.4 years (SD=9.0). Regarding the field of activity, 11 (34.4%) of the experts worked in healthcare; 10 (31.3%) in teaching, and 9 (28.1%) in research. Out of the 32 participants, 15 (46.9%) worked in the field of infectious diseases, 8 (26.7%) had a doctorate as their highest level of education; 16 (53.3%) had knowledge of HIV/AIDS acquired at work; and 6 (20.0%) through publications in the field. Among the participants, 28 (87.5%) worked and provided daily guidance to users on PrEP. The educational booklet validation questionnaire comprises 55 attributes, each with statements evaluated by experts, ranging from “Totally agree” to “Totally disagree”. Validation of an attribute requires that the sum of the responses “Totally agree” and “Agree” be greater than 80% of the total responses. The attributes are distributed into three domains: “Overall evaluation”, “Content” and “Aesthetic and visual quality”. The “Overall evaluation” domain includes 10 attributes, listed in Table 1, along with their respective I-CVIs.

**Table 1:** Overall evaluation of the educational booklet by health experts (N=32), Ribeirão Preto, São Paulo, Brazil, 2024

Attributes evaluated	SA	A	DK	D	SD	I-CVI
General assessment						
The booklet is described in a way that allows understanding for PrEP usage.	20	12	-	-	-	1.00
The booklet is described in a way that allows understanding of the effectiveness of PrEP.	18	14	-	-	-	1.00
The booklet is described in a way that allows understanding PrEP’s benefits.	19	13	-	-	-	1.00
The booklet is described in a way that allows understanding of the possible side effects of PrEP.	19	11	1	1	-	0.94
The booklet is described in a way that allows understanding of the eligibility criteria for PrEP use.	17	14	1	-	-	0.97
The booklet is described in a way that allows understanding of adherence to PrEP.	20	12	-	-	-	1.00
The booklet is described in such a way as to allow the understanding of facilitating strategies for monitoring PrEP.	20	12	-	-	-	1.00
The booklet is described in such a way as to allow the understanding of facilitating strategies for monitoring PrEP.	17	12	2	1	-	0.91
It is consistent with the needs of people using PrEP	17	14	1	-	-	0.97
It is consistent with the needs of people using PrEP	22	9	-	1	-	0.97
S-CVI/Ave						0.97

* *Number of experts who chose the option: SA - Strongly agree; A - Agree; DK - Don’t know; D - Disagree; SD - Strongly disagree. I-CVI = Item-Level Content Validity Index. S-CVI/Ave = Scale-level Content Validity Index, Average calculation method.*

The content and written language domain (Table 2), composed of 22 attributes, achieved an S-CVI/Ave of 0.96, ranging from 0.88 to 1.00. After analyzing the experts’ suggestions, it was identified the need to reinforce the possible contraindications to the concomitant use of PrEP, emphasize that it does not prevent other STIs and improve the exposure on the use of internal and external condoms.

**Table 2:** Evaluation of content and written language of the educational booklet by health experts (N=32), Ribeirão Preto, São Paulo, Brazil, 2024

Attributes evaluated	SA	A	DK	D	SD	I-CVI
Content						
The booklet contains all the information necessary for guidance on the use of PrEP.	17	12	1	2	-	0.91
The booklet contains all the information necessary for guidance on the effectiveness of PrEP.	19	12	-	1	-	0.97
The booklet contains all the information necessary for guidance on the benefits of PrEP.	18	14	-	-	-	1.00
The booklet contains all the information necessary for guidance on the possible side effects of PrEP.	21	10	-	1	-	0.97
The booklet contains all the information necessary for guidance on the eligibility criteria for PrEP.	17	12	2	1	-	0.91
The booklet contains all the information necessary for guidance on adherence to PrEP treatment.	19	12	-	1	-	0.97
The booklet contains all the information necessary for guidance on strategies to facilitate PrEP monitoring.	17	15	-	-	-	1.00
The sequence of the booklet's content is appropriate.	17	14	-	1	-	0.97
The content allows for understanding the topic.	19	13	-	-	-	1.00
The content covered is in accordance with current knowledge.	19	12	-	1	-	0.97
The content allows for understanding the use of PrEP.	16	16	-	-	-	1.00
The content allows for understanding the effectiveness of PrEP.	17	14	-	1	-	0.97
The content allows for understanding the benefits of PrEP.	21	11	-	-	-	1.00
The content allows for understanding the possible side effects of PrEP.	18	13	-	1	-	0.97
The content is appropriate for the target audience (adults using PrEP) PrEP).	14	14	1	3	-	0.88
The content allows the understanding of adherence to PrEP treatment.	17	14	-	1	-	0.97
The content allows the understanding of strategies that facilitate PrEP monitoring.	21	10	-	1	-	0.97
The guidelines presented are relevant.	19	13	-	-	-	1.00
The guidelines presented were addressed correctly.	15	14	-	3	-	0.91
The information is appropriate for the target audience.	14	17	-	1	-	0.97
The information is presented in a context that is relevant to the target audience	14	15	-	3	-	0.91
The booklet contains all the information necessary for using PrEP	14	16	-	1	1	0.94
S-CVI/Ave						0.96

* *Number of experts who chose the option: SA - Strongly agree; A - Agree; DK - Don’t know; D - Disagree; SD - Strongly disagree. I-CVI = Item-Level Content Validity Index. S-CVI/Ave = Scale-level Content Validity Index, Average calculation method.*

The last domain assessed by the experts was the aesthetic and visual quality of the educational booklet, consisting of 8 attributes. The objective was to verify whether the material is attractive and arouses interest through its colors and images. The domain obtained a total CVI of 0.91, meeting the minimum value required for approval in all attributes, as shown in Table 3. However, the experts suggested improvements, such as increasing the font size, including more diverse characters and making the booklet more colorful and attractive, especially for teenagers.

**Table 3:** Evaluation of the aesthetic and visual quality of the educational booklet by health experts (N=32), Ribeirão Preto, São Paulo, Brazil, 2024

Attributes evaluated	SA	A	DK	D	SD	I-CVI
Aesthetic and visual quality						
The visual aspect of the booklet is interesting.	18	11	-	3	-	0.91
The visual aspect of the booklet motivates its reading.	18	12	-	1	1	0.94
The booklet has a satisfactory aesthetic/layout.	18	9	3	2	-	0.84
The format and size of the letters are satisfactory.	14	14	1	3	-	0.88
The infographics created retain the written guidelines.	19	11	1	1	-	0.94
The quality of the infographics is satisfactory.	18	13	1	-	-	0.97
The colors used do not hinder reading.	20	9	2	1	-	0.91
The layout favors understanding of the message.	20	10	-	2	-	0.94
S-CVI/Ave						0.91

* *Nº de especialistas que escolheram a opção: CT - Concordo totalmente; C - Concordo; NS - Não sei; D - Discordo; DT - Discordo totalmente. I-CVI = Item-Level Content Validity Index. S-CVI/Ave = Scale-level Content Validity Index, Average calculation method.*

The experts’ suggestions were accepted and a final version of the educational booklet was prepared taking into account the modifications suggested by experts as relevant to composing the educational booklet.

The overall Content Validity Index (S-CVI/UA) of the validation with the experts was given by the average of the total IVC, that is, the sum of the S-CVI/Ave of each domain divided by the number of domains (4), whose result was 0.94, thus validating the educational booklet through all the Content Validity Index (by attributes, domain and overall) reaching a value equal to or greater than 0.80.

### Validation of the educational booklet by the target audience

The validation with the target audience included 13 PrEP users, with an average age of 36.7 years, all male, cisgender and men who have sex with men (MSM). The majority of participants (84.6%) were single, and the majority had completed college (38.5%) and were employed (46.2%). All participants (100%) were current PrEP users, received follow-up guidance and intended to continue using it. The results regarding the organization of the material, writing style, appearance and motivation are presented in Table 4.

**Table 4:** Evaluation of the organization, writing style, appearance and motivation of the educational booklet by the target audience (n=13), Ribeirão Preto, São Paulo, Brazil, 2024

Attributes evaluated	CT/C	D/DT	IVC
Organization			
The information provided in the booklet is easy to understand.	13	-	1.00
The information provided in the booklet is important.	13	-	1.00
The information in the booklet helps me decide whether or not I want to use PrEP.	12	1	0.92
Writing and appearance			
I liked how the content of the booklet was presented.	13	-	1.00
The images help to understand the content of the booklet.	13	-	1.00
The colors used in the booklet are attractive.	11	2	0.85
The booklet makes it possible to understand what PrEP is.	12	1	0.92
The booklet makes it possible to understand the benefits of using PrEP.	12	1	0.92
The booklet helped me understand the importance of professional monitoring while using PrEP.	12	1	0.92
The booklet makes it possible to understand the possible side effects of PrEP.	11	2	0.85
The booklet helps to understand some precautions that should be taken while using PrEP.	12	1	0.92
The booklet allows me to know where to get PrEP.	12	1	0.92
The booklet makes it possible to understand who can use PrEP.	13	-	1.00
Motivation			
The booklet can be a facilitator in the search for PrEP.	12	1	0.92
The booklet made me realize that I meet the criteria for indication for PrEP.	13	-	1.00
The information in this booklet made me interested in seeking/starting PrEP.	12	1	0.92
The information in this booklet can help improve adherence to PrEP.	13	-	1.00
I know someone who would benefit from reading this booklet.	13	-	1.00
This booklet can be a complementary tool in sexual education activities for people over 16 years old.	13	-	1.00

* *Number of experts who chose the option: SA - Strongly agree; A - Agree; DK - Don’t know; D - Disagree; SD - Strongly disagree. I-CVI=Item-Level Content Validity Index, 2024.*

Two negative responses were highlighted in the target audience evaluations, resulting in a CI of 0.85 in the written category. Participants were invited to provide suggestions and comments, which addressed the need for details on where to seek PrEP in the SUS and in the private system. Positive observations were also recorded, praising the easy understanding and relevance of the booklet for those who want or use PrEP.

## DISCUSSION

This study describes the development and validation of an educational booklet for HIV Pre-Exposure Prophylaxis (PrEP) users, with the aim of supporting their prevention and self-care strategies. Developed based on the literature and validated by health professionals and PrEP users, this educational tool aims to promote the conscious use of PrEP and expand its reach. The use of educational technologies such as this booklet is essential to increase knowledge, promote autonomy and facilitate self-care, especially in a context where verbal guidance predominates, ensuring that the educational needs of PrEP users are met in an individualized and person-centered manner.

In the current context, the fight against AIDS has continued for more than four decades, and it is still considered a disease full of social stigmas, especially related to sexual minorities, such as LGBTQIA+^(20)^. Over this time, there have been great advances in the areas of health and technology, facilitating access to new information. In fact, greater access to information by the population directly impacts public health issues, since health literacy provides individuals with the ability to exercise autonomy over their health processes and strengthen decision-making in a more conscious and informed manner^(21)^.

Controlling HIV transmission is still a major global challenge, and as such, several goals have been established in an attempt to achieve it. An example of this is one of the SDG goals, specifically the goal of health and well-being, which aims to eliminate AIDS by 2030^(2)^. Thus, one way to help achieve the eradication of AIDS is through health literacy among the population regarding prevention methods. For this process to occur, teaching materials that facilitate the understanding of information are necessary^(22)^.

Educational technologies have played a fundamental role in health education, especially by directing strategies to raise awareness of issues that deal with certain topics; through playful and interactive exposure to the target audience; It can be written with easy understanding in mind, using simplified language and including relevant information^(24)^. In the case of the booklet developed during this study, it serves the purpose of raising awareness among the target audience about the struggles to eradicate AIDS, prevention and greater safety, with the guarantee of reliable, up-to-date evidence written in an accessible manner, both in terms of vocabulary and easy interpretation.

An educational booklet, whether in printed or online format, helps to disseminate knowledge with a broad reach to individuals in both modalities. The educational booklet that aims at health literacy, containing easy-to-understand texts and self-explanatory images that facilitate the understanding of the material, especially, has the potential to reach vulnerable populations, since they do not need adjacent support, such as an internet connection and electronic devices for reading. Thus, it is a great tool for health professionals, in the context of confronting and implementing health prevention strategies^(9)^.

The educational booklet addressed the main topics identified in the scientific evidence related to PrEP, such as its functioning, indication, efficacy, benefits and side effects, obtaining a Global Content Validity Index (S-CVI/UA) of 0.94, above the target considered for approval, which is an index higher than 0.80. Thus, it is considered valid by health experts and the target audience for future use as an educational technology with the population, since the booklet was developed in order to provide updated information on PrEP in an accessible way.

Validation with the target audience revealed a generally positive assessment of the booklet in relation to its writing, appearance and motivation. Despite some opinions about the colors, most users considered it useful for promoting health education beyond the specialized service. The colors were adjusted to better suit the users’ profile, and the writing of side effects was simplified. This validation is crucial to identify gaps in understanding and ensure that the educational material is accessible and relevant to different levels of health literacy. An organized and attractive presentation of the content is essential for effective reading, aligned with the interests and needs of readers.

A well-developed educational booklet is a valuable resource in health education strategies, facilitating understanding, being accessible and reinforcing the guidelines provided verbally by the health team. The printed format offers the advantage of being able to be consulted later, serving as support for consultations and readings over time and favoring communication with the health team. It is also worth noting that the booklet does not replace the health professional in health education actions; however, it can facilitate communication and encourage self-care^(24)^.

### Study limitations

The booklet was validated with young adults with a high level of education who were already linked to a specialized service, excluding groups with low levels of education. Despite this, all stages were completed. The validated population reflects real PrEP users in the country. It is suggested that other educational technologies, such as games and videos, can be created to reach different population groups.

### Contributions to the area

The development and validation of a new booklet for PrEP users represents an important educational technology that is low cost and easy to implement in health services. It can reach a wide audience and be used in educational interventions as a teaching-learning strategy by nursing staff and other health professionals. This study addresses the main topics to be included in the guidelines for the use of PrEP, highlighting the importance of nurses in the health education process.

## CONCLUSIONS

The educational booklet on PrEP was prepared in five topics and validated by experienced professionals and users. Its final printed version can be used as a complementary resource to the guidance provided by nurses and doctors during specialized monitoring, thus helping to ensure universal access to quality health services. Such access is crucial to achieving the third of the UN Sustainable Development Goals. In addition, the booklet aims to strengthen health literacy about PrEP, a free HIV prevention method provided by the SUS. This empowers individuals to make informed decisions about their health, thus promoting their empowerment and autonomy.

## Data Availability

Not applicable.
