# Supplementary material for: Construction and validation of an educational booklet on HIV pre-exposure prophylaxis
Source: Rev Bras Enferm. 2025 Sep 1;78(Suppl 1):e20240245. doi: 10.1590/0034-7167-2024-0245 (PMC12407884; doi:10.1590/0034-7167-2024-0245)
Supplement: Translation

# Translation: Construção e validação de cartilha educativa sobre a profilaxia pré-exposição ao HIV

**DOI:** 10.1590/0034-7167-2024-0245pt

**Authors:** Marina Andreoli Trigo, Karyanna Alves de Alencar Rocha, Marcela Antonini, Daniel de Macêdo Rocha, Henrique Ciabotti Elias, Renata Karina Reis

**Affiliations:** I Universidade de São Paulo. Ribeirão Preto, São Paulo, Brasil

**Keywords:** Tecnologia Educacional, Prevenção de Doenças, HIV, Profilaxia Pré-Exposição, Enfermagem.

## Abstract

**Objetivos:**

construir e validar uma cartilha educativa sobre a Profilaxia Pré-Exposição (PrEP) ao HIV.

**Métodos:**

estudo de validação, quantitativo, voltado para a construção e validação de cartilha educativa, por especialistas e público-alvo. Para a validação com especialistas, utilizou-se o Índice de Validação de Conteúdo Educativo em saúde desenvolvido em ambiente virtual, e com o público-alvo utilizou-se um questionário com questões relacionadas à organização, estilo da escrita, aparência e motivação.

**Resultados:**

participaram do estudo 32 especialistas e 13 usuários de PrEP. A análise foi estruturada nos princípios da estatística descritiva, considerados satisfatórios os domínios que apresentaram Índice de Validade de Conteúdo igual ou superior à 0,80. A versão final do material foi disponibilizada no formato online e impresso.

**Conclusões:**

a cartilha educativa foi desenvolvida e validada por especialistas e público-alvo, servindo como uma ferramenta educativa de apoio para o autocuidado e conscientização da prevenção do HIV por usuários de PrEP.

## INTRODUÇÃO

A infecção pelo vírus da imunodeficiência humana (HIV) continua a ser um desafio global de saúde pública, com 85,6 milhões de pessoas infectadas e 40,4 milhões de mortes relacionadas à AIDS registradas desde o surgimento da pandemia. Em 2022, 1,3 milhão de novos casos de HIV foram diagnosticados, resultando em 630 mil mortes por aids^(1)^.

Diante da grande preocupação com o controle da transmissão do vírus, durante a cúpula das Nações Unidas sobre Objetivos de Desenvolvimento Sustentável (ODS), em setembro de 2015, foram estabelecidos 17 objetivos: dentre eles, o objetivo nº 3 é descrito como saúde e bem-estar, em que engloba metas relacionadas ao tema. Tendo como uma de suas metas a erradicação da aids e outras doenças negligenciadas. A eliminação da aids é um objetivo importante para a saúde pública, uma vez que ela ceifou cerca de 630 mil vidas em 2022, segundo estatísticas da UNAIDS, demonstrando que mesmo com os avanços alcançados pela ciência e pelos esforços conjuntos das nações, objetivando interromper a cadeia de transmissão e diminuir o número de novas infecções, ainda ocorrem perdas significativas^(2)^.

A Profilaxia Pré-Exposição (PrEP) foi implementada no Brasil em 2017 para populações expostas ao HIV, com critérios de indicação ampliados para toda a população com mais de 15 anos e peso superior a 35 kg, incluindo casais sorodiferentes e pessoas que usam drogas injetáveis^(3,4)^. A PrEP é uma terapia medicamentosa diária com antirretrovirais por via oral, segura, com poucos efeitos colaterais e com eficácia de proteção comprovada superior a 90% quando utilizada corretamente^(4)^. Apesar de sua eficácia, o conhecimento sobre a PrEP na população ainda é limitado ou lacunar, o que dificulta sua adoção generalizada^(5)^.

Um dos principais desafios atuais enfrentados pelos profissionais de saúde é a comunicação acessível de informações sobre a Profilaxia Pré-Exposição (PrEP), atendendo às necessidades e dúvidas tanto dos indivíduos quanto da comunidade de forma clara e objetiva^(6)^. A demanda por educação em saúde é generalizada, e constantemente discute-se maneiras de fornecer informações e atualizações científicas de forma acessível à população, garantindo que ela possa compreender e utilizar conteúdo baseados em evidências confiáveis. Além disso, a forma como essas informações são disponibilizadas influencia diretamente o processo de aprendizagem e, consequentemente, a saúde dos indivíduos^(7,8)^.

A tecnologia educacional é uma ferramenta crucial na enfermagem para promover estratégias de prevenção em saúde, incluindo a conscientização sobre o HIV e métodos de prevenção, como a prevenção combinada. Destaca-se, também, a importância de indivíduos com HIV manterem sua carga viral indetectável por meio do tratamento, interrompendo assim o ciclo de transmissão^(9)^.

Considerando a problemática da dificuldade de acesso da população a informações claras e objetivas sobre a PrEP, bem como a necessidade de fortalecer as estratégias de prevenção à aids, conforme delineado no terceiro dos Objetivos de Desenvolvimento Sustentável (ODS), é imperativo criar um material de fácil acesso e entendimento para promover a educação em saúde. Dessa forma, este estudo teve o objetivo de construir e validar uma cartilha educativa sobre a Profilaxia Pré-exposição (PrEP) ao HIV.

## OBJETIVOS

Construir e validar uma cartilha educativa sobre a Profilaxia Pré-exposição (PrEP) ao HIV.

## MÉTODOS

### Aspectos éticos

O estudo foi aprovado pelo Comitê de Ética em Pesquisa, sendo assegurado o cumprimento às recomendações da Resolução Nº 466/12. Foi conduzido por meio da Plataforma *Research Electronic Data Capture* (REDCAp)^(10)^ com abrangência nacional.

### Desenho, período e local do estudo

Trata-se de um estudo de validação, quantitativo, desenvolvido em três etapas: construção da cartilha educativa, validação de conteúdo com especialistas e validação com o público-alvo, realizada no período de dezembro de 2022 a fevereiro de 2024. Este estudo segue as diretrizes do *Revised Standards for Quality Improvement Reporting Excellence* (SQUIRE 2.0)^(11-14)^.

### Construção da cartilha educativa

A etapa da pré-produção da cartilha educativa consistiu em uma revisão de literatura sobre a Profilaxia de Pré-exposição ao HIV articulada com as recomendações do Protocolo clínico e diretrizes terapêuticas para Profilaxia Pré-exposição (PrEP) (2022) de risco à infecção pelo HIV e evidências científicas internacionais atuais, no período de maio a dezembro de 2022^(3)^. As publicações passaram por leitura crítica a fim de se extrair informações úteis para constar na cartilha educativa, sendo definidos os principais tópicos e seus objetivos.

A produção da cartilha educativa incluiu diagramação e infográficos para facilitar a compreensão. Um designer especializado em materiais educativos em saúde foi contratado para este fim. O processo ocorreu de dezembro de 2022 a julho de 2023, seguindo as diretrizes propostas por Moreira (2003) para criar um material educativo eficaz em termos de linguagem, layout e ilustrações.

### Amostra e critérios de elegibilidade para validação

Para identificação e seleção dos especialistas, fez-se necessário o emprego de critérios que evidenciam a expertise de conhecimento específico em saúde sobre o tema do estudo, conforme critérios adotados por Fehring (1994)^(15)^. Para validar a cartilha educativa, foram convidados usuários de PrEP em acompanhamento no Centro de Referência em Especialidades Central de Ribeirão Preto, entre outubro e dezembro de 2023. Os critérios de inclusão foram: idade acima de 18 anos, estabilidade clínica, lucidez e disposição para ler a cartilha. A coleta de dados foi realizada em uma sala do serviço para garantir o sigilo. Pacientes com incapacidade visual ou cognitiva foram excluídos. A seleção dos participantes foi feita de forma consecutiva durante o período do estudo. O Consentimento Livre e Esclarecido foi obtido de todos os indivíduos envolvidos no estudo por meio online.

### Validação dos especialistas

A etapa de validação foi realizada de forma virtual, refletindo tendências de utilizar a via digital para facilitar a realização de estudos empíricos, servindo como meio para a coleta e divulgação de resultados^(16,17)^. O recrutamento empregou o método *Snowball*, como descrito por Fernandes e Carvalho (2003)^(18)^, adaptado para o uso em redes sociais virtuais como meio de coleta de dados e disseminação de resultados científicos.

A avaliação da cartilha educativa foi conduzida através de um questionário autoaplicável e virtual, inserido na plataforma REDCap^(12)^ e adaptado do modelo de Validação de Conteúdo Diagnóstico de Enfermagem proposto por Fehring (1994)^(15)^. O questionário utilizou uma escala do tipo Likert para analisar a cartilha em diferentes aspectos, desde seus objetivos até sua estrutura, apresentação e relevância. Os participantes puderam fornecer sugestões e observações sobre os atributos avaliados, incluindo a identificação de erros ou a solicitação de acréscimo de conteúdo relevante.

### Validação do público-alvo

Durante as consultas de acompanhamento da PrEP no serviço especializado, uma das pesquisadoras do Núcleo de aids e DST (NAIDST) abordava os usuários e convidava-os para participar da validação. Após aceitação, os participantes recebiam um questionário semiestruturado sobre dados sociodemográficos, um questionário para validação elaborado pelos pesquisadores e a cartilha educativa em formato virtual. O questionário de validação, adaptado de um estudo anterior, continha 20 questões agrupadas em organização, escrita, aparência e motivação. As respostas eram categorizadas como concordo totalmente/concordo (respostas positivas) e discordo/discordo totalmente (respostas negativas).

### Análise dos resultados e estatística

Para analisar as respostas dos especialistas, utilizou-se o Índice de Validação de Conteúdo (IVC)^(17)^, que mede a proporção de concordância entre os avaliadores. O consenso foi considerado satisfatório quando o CVI foi igual ou superior a 80%. O IVC permitiu verificar a concordância dos especialistas em relação à representatividade do conteúdo. Já para avaliar as respostas dos usuários, utilizou-se o Índice de Concordância (IC)^(17-21)^, que calcula o número de concordâncias dividido pelo total de avaliações. Uma concordância superior ou igual a 0,8 foi considerada satisfatória. Os dados sociodemográficos foram analisados de forma descritiva, apresentando-se em tabelas, e contribuíram para possíveis modificações na cartilha, resultando em sua versão final. O estudo recebeu aprovação do Comitê de Ética em Pesquisa da Escola de Enfermagem de Ribeirão Preto, Universidade de São Paulo.

## RESULTADOS

### Construção da cartilha educativa

A cartilha educativa intitulada *Profilaxia Pré-Exposição ao Vírus da Imunodeficiência Humana: mais uma opção de prevenção ao HIV* (2024) possui 16 páginas, dividida em cinco tópicos contando com ilustrações e pequenos textos informativos. Para a construção da cartilha educativa foram reunidas as informações básicas sobre a PrEP^(22)^, a partir do Protocolo Clínico e Diretrizes Terapêuticas para Profilaxia Pré-Exposição (PrEP) de Risco à Infecção pelo HIV (2022), e contratado um *designer* profissional considerando a distribuição de elementos de forma coesiva, utilizando cores associadas ao tema de saúde e agradáveis esteticamente conforme observado na Figura 1.

**Figura 1:** Cartilha educativa: Profilaxia Pré-Exposição ao Vírus da Imunodeficiência Humana

*Fonte: Cartilha educativa sobre PrEP, 2024.*

O primeiro tópico da cartilha educativa, intitulado “Profilaxia Pré-Exposição ao HIV, como funciona?”, descreve como os medicamentos funcionam e a indicação da PrEP. O segundo tópico, “Eficácia e Benefício”, aborda questões como quando a PrEP começa a fazer efeito, o acompanhamento necessário, benefícios e efeitos colaterais. O terceiro tópico, “Quero utilizar”, responde quem pode utilizar a PrEP, como iniciar e continuar seu uso, com base no protocolo brasileiro. O conteúdo foi adaptado para ser acessível, livre de termos técnicos complexos e lúdico, visando alcançar e ser compreendido pela população em diferentes contextos. O quarto tópico, “Estratégias para não esquecer de tomar”, oferece seis orientações simples para melhorar a adesão à PrEP, como anotações e o uso de despertadores. Por fim, o quinto tópico, “Onde buscar mais informações”, lista sites oficiais e endereços eletrônicos para obter informações adicionais sobre a PrEP^(9)^.

Os infográficos foram inseridos ao longo do texto para facilitar a leitura e foram elaborados elementos que buscam reforçar a mensagem escrita. A cor predominante utilizada foi a cor azul, que Fernandes e Araújo (2018)^(23)^ descrevem como uma cor capaz de trazer a sensação de calma e relaxamento e afastar a sensação de pressa e agitação que o indivíduo carrega todos os dias.

### Validação da cartilha educativa pelos especialistas

A etapa de validação da cartilha educativa contou com a participação de 32 especialistas. Estes eram predominantemente graduados em Enfermagem 29 (93,5%), do sexo feminino 18 (56,3%) de regiões distintas do país. A média do tempo de atuação na área foi de 8,4 anos (DP=9,0). Com relação à área de atuação, 11 (34,4%) dos especialistas pertencem à assistência; 10 (31,3%) à docência, e 9 (28,1%) à pesquisa. Dos 32 participantes, 15 (46,9%) atuam na área da infectologia, 8 (26,7%) possuem doutorado como maior nível de formação; 16 (53,3%) possuem conhecimento em HIV/aids adquirido no local de trabalho; e 6 (20,0%) através de publicações na área. Entre os participantes, 28 (87,5%) atuam e realizam orientações diariamente aos usuários sobre a PrEP.

O questionário de validação da cartilha educativa compreende 55 atributos, cada um com afirmações avaliadas pelos especialistas, variando de “Concordo totalmente” a “Discordo totalmente”. A validação de um atributo requer que a soma das respostas “Concordo totalmente” e “Concordo” seja superior a 80% do total das respostas. Os atributos são distribuídos em três domínios: “Avaliação geral”, “Conteúdo” e “Qualidade estética e visual”. O domínio de “Avaliação geral” inclui 10 atributos, listados na Tabela 1, juntamente com seus respectivos I-CVIs.

**Tabela 1:** Avaliação geral da cartilha educativa pelos especialistas na área da saúde (N=32), Ribeirão Preto, São Paulo, Brasil, 2024

Atributos avaliados	CT	C	NS	D	DT	I-CVI
Avaliação geral						
A cartilha está descrita de forma a permitir a compreensão do uso da PrEP	20	12	-	-	-	1,00
A cartilha está descrita de forma a permitir a compreensão da eficácia da PrEP	18	14	-	-	-	1,00
A cartilha está descrita de forma a permitir a compreensão dos benefícios da PrEP	19	13	-	-	-	1,00
A cartilha está descrita de forma a permitir a compreensão dos possíveis efeitos colaterais da PrEP	19	11	1	1	-	0,94
A cartilha está descrita de forma a permitir a compreensão dos critérios de elegibilidade para uso da PrEP	17	14	1	-	-	0,97
A cartilha está descrita de forma a permitir a compreensão sobre a adesão à PrEP	20	12	-	-	-	1,00
A cartilha está descrita de forma a permitir a compreensão de estratégias facilitadoras para o acompanhamento da PrEP	20	12	-	-	-	1,00
A cartilha está descrita de forma a permitir a compreensão de estratégias facilitadoras para o acompanhamento da PrEP	17	12	2	1	-	0,91
É coerente com as necessidades das pessoas em uso da PrEP	17	14	1	-	-	0,97
É coerente com as necessidades das pessoas em uso da PrEP	22	9	-	1	-	0,97
S-CVI/Ave						0,97

* *Nº de especialistas que escolheram a opção: CT - Concordo totalmente; C - Concordo; NS - Não sei; D - Discordo; DT - Discordo totalmente. I-CVI = Item-Level Content Validity Index. S-CVI/Ave = Scale-level Content Validity Index, Average calculation method.*

O domínio de conteúdo e linguagem escrita (Tabela 2), composto por 22 atributos, alcançou um S-CVI/Ave de 0,96, variando de 0,88 a 1,00. Após a análise das sugestões dos especialistas, identificou-se a necessidade de reforçar as possíveis contraindicações ao uso concomitante da PrEP, enfatizar que ela não previne outras IST e aprimorar a exposição sobre o uso de preservativos internos e externos.

**Tabela 2:** Avaliação de conteúdo e linguagem escrita da cartilha educativa pelos especialistas na área da saúde (N=32), Ribeirão Preto, São Paulo, Brasil, 2024

Atributos avaliados	CT	C	NS	D	DT	I-CVI
Conteúdo						
A cartilha dispõe de todas as informações necessárias para orientação sobre o uso da PrEP	17	12	1	2	-	0,91
A cartilha dispõe de todas as informações necessárias para orientação sobre a eficácia da PrEP	19	12	-	1	-	0,97
A cartilha dispõe de todas as informações necessárias para orientação sobre os benefícios da PrEP	18	14	-	-	-	1,00
A cartilha dispõe de todas as informações necessárias para orientação sobre os possíveis efeitos colaterais da PrEP	21	10	-	1	-	0,97
A cartilha dispõe de todas as informações necessárias para orientação sobre os critérios de elegibilidade da PrEP	17	12	2	1	-	0,91
A cartilha dispõe de todas as informações necessárias para orientação sobre a adesão ao tratamento da PrEP	19	12	-	1	-	0,97
A cartilha dispõe de todas as informações necessárias para orientação sobre estratégias facilitadoras para o acompanhamento da PrEP	17	15	-	-	-	1,00
A sequência do conteúdo da cartilha está adequada	17	14	-	1	-	0,97
O conteúdo permite a compreensão do tema	19	13	-	-	-	1,00
Os conteúdos abordados estão de acordo com o conhecimento atual	19	12	-	1	-	0,97
O conteúdo permite a compreensão do uso da PrEP	16	16	-	-	-	1,00
O conteúdo permite a compreensão da eficácia da PrEP	17	14	-	1	-	0,97
O conteúdo permite a compreensão dos benefícios da PrEP	21	11	-	-	-	1,00
O conteúdo permite a compreensão dos possíveis efeitos colaterais da PrEP	18	13	-	1	-	0,97
O conteúdo está adequado ao público-alvo (pessoas adultas em uso da PrEP)	14	14	1	3	-	0,88
O conteúdo permite a compreensão sobre a adesão ao tratamento da PrEP	17	14	-	1	-	0,97
O conteúdo permite a compreensão de estratégias facilitadoras para o acompanhamento da PrEP	21	10	-	1	-	0,97
As orientações apresentadas são pertinentes	19	13	-	-	-	1,00
As orientações apresentadas foram abordadas corretamente	15	14	-	3	-	0,91
As informações são apropriadas ao público-alvo	14	17	-	1	-	0,97
As informações são apresentadas em um contexto pertinente ao público-alvo	14	15	-	3	-	0,91
A cartilha dispõe de todas as informações necessárias para o uso da PrEP	14	16	-	1	1	0,94
S-CVI/Ave						0,96

* *Nº de especialistas que escolheram a opção: CT - Concordo totalmente; C - Concordo; NS - Não sei; D - Discordo; DT - Discordo totalmente. I-CVI = Item-Level Content Validity Index. S-CVI/Ave = Scale-level Content Validity Index, Average calculation method.*

O último domínio avaliado pelos especialistas foi a qualidade estética e visual da cartilha educativa, composto por 8 atributos. O objetivo era verificar se o material é atrativo e desperta interesse por meio de suas cores e imagens. O domínio obteve um IVC total de 0,91, atendendo ao valor mínimo necessário para aprovação em todos os atributos, conforme demonstrado na Tabela 3. No entanto, os especialistas sugeriram melhorias, como aumentar o tamanho da letra, incluir mais diversidade de personagens e tornar a cartilha mais colorida e atrativa, especialmente para adolescentes.

**Tabela 3:** Avaliação da qualidade estética e visual da cartilha educativa pelos especialistas na área da saúde (N=32), Ribeirão Preto, São Paulo, Brasil, 2024

Atributos avaliados	CT	C	NS	D	DT	I-CVI
Qualidade estética e visual						
O aspecto visual da cartilha é interessante	18	11	-	3	-	0,91
O aspecto visual da cartilha é motivador para a leitura	18	12	-	1	1	0,94
A cartilha apresenta uma estética/layout satisfatório	18	9	3	2	-	0,84
O formato e tamanho das letras estão satisfatórios	14	14	1	3	-	0,88
Os infográficos elaborados retêm as orientações por escrito	19	11	1	1	-	0,94
A qualidade dos infográficos é satisfatória	18	13	1	-	-	0,97
As cores utilizadas não atrapalham a leitura	20	9	2	1	-	0,91
A diagramação favorece o entendimento da mensagem	20	10	-	2	-	0,94
S-CVI/Ave						0,91

* *Nº de especialistas que escolheram a opção: CT - Concordo totalmente; C - Concordo; NS - Não sei; D - Discordo; DT - Discordo totalmente. I-CVI = Item-Level Content Validity Index. S-CVI/Ave = Scale-level Content Validity Index, Average calculation method.*

As sugestões dos especialistas foram acatadas e uma versão final da cartilha educativa foi elaborada levando em consideração as modificações sugeridas por especialistas como relevantes para compor a cartilha educativa.

O Índice de Validade de Conteúdo (S-CVI/UA) global da validação com os especialistas se deu pela média de IVC total, ou seja, a somatória dos S-CVI/Ave de cada domínio dividido pelo número de domínios (4), cujo resultado foi 0,94, validando, dessa forma, a cartilha educativa mediante todos os Índice de Validade de Conteúdo (por atributos, domínio e global) sendo alcançado um valor igual ou maior a 0,80.

### Validação da cartilha educativa pelo público-alvo

A validação junto ao público-alvo contou com 13 usuários de PrEP, com idade média de 36,7 anos, todos do sexo masculino, cisgênero e homens que fazem sexo com homens (HSH). A maioria dos participantes (84,6%) era solteira, e a maioria possuía graduação completa (38,5%) e vínculo empregatício (46,2%). Todos os participantes (100%) eram usuários atuais da PrEP, recebiam orientações de acompanhamento e pretendiam continuar utilizando. Os resultados sobre a organização do material, estilo da escrita, aparência e motivação estão apresentados na Tabela 4.

**Tabela 4:** Avaliação da organização, escrita, aparência e motivação da cartilha educativa pelo público-alvo (n=13), Ribeirão Preto, São Paulo, Brasil, 2024

Atributos avaliados	CT/C	D/DT	IVC
Organização			
As informações disponibilizadas na cartilha são fáceis de entender.	13	-	1,00
As informações disponibilizadas na cartilha são importantes	13	-	1,00
As informações da cartilha ajudam a decidir se quero ou não utilizar a PrEP	12	1	0,92
Escrita e aparência			
Eu gostei como o conteúdo da cartilha foi apresentado.	13	-	1,00
As imagens ajudam a entender o conteúdo da cartilha.	13	-	1,00
As cores utilizadas na cartilha são atrativas.	11	2	0,85
A cartilha possibilita compreender o que é a PrEP	12	1	0,92
A cartilha possibilita compreender os benefícios de usar a PrEP.	12	1	0,92
A cartilha me ajudou a compreender a importância do acompanhamento profissional durante o uso da PrEP.	12	1	0,92
A cartilha possibilita a compreensão de possíveis efeitos colaterais da PrEP	11	2	0,85
A cartilha ajuda a compreender alguns cuidados que devem ser feitos durante o uso da PrEP	12	1	0,92
A cartilha permite que eu saiba onde buscar a PrEP	12	1	0,92
A cartilha permite compreender quem pode utilizar a PrEP	13	-	1,00
Motivação			
A cartilha pode ser um facilitador da busca pela PrEP	12	1	0,92
A cartilha me fez perceber que eu me encaixo nos critérios de indicação para a PrEP.	13	-	1,00
As informações desta cartilha me causaram interesse em buscar/ iniciar a PrEP	12	1	0,92
As informações desta cartilha que podem ajudar a melhorar a adesão a PrEP.	13	-	1,00
Eu conheço alguém que se beneficiaria lendo essa cartilha	13	-	1,00
Essa cartilha pode ser uma ferramenta complementar em atividades de educação sexual para maiores de 16 anos.	13	-	1,00

* *Nº de especialistas que escolheram a opção: CT - Concordo totalmente; C - Concordo; D - Discordo; DT - Discordo totalmente. I-CVI = Item-Level Content Validity Index, 2024.*

Duas respostas negativas foram destacadas nas avaliações do público-alvo, resultando em um IC de 0,85 na categoria escrita. Os participantes foram convidados a fornecer sugestões e comentários, que abordaram a necessidade de detalhes sobre onde buscar a PrEP no SUS e no sistema privado. Observações positivas também foram registradas, elogiando o fácil entendimento e a pertinência da cartilha para quem deseja ou utiliza a PrEP.

## DISCUSSÃO

Este estudo descreve a construção e validação de uma cartilha educativa destinada a usuários da Profilaxia Pré-Exposição (PrEP) ao HIV, com o objetivo de apoiar suas estratégias de prevenção e autocuidado. Desenvolvida com base na literatura e validada por profissionais de saúde e usuários de PrEP, essa ferramenta educativa visa promover o uso consciente da PrEP e ampliar seu alcance. O uso de tecnologias educativas como essa cartilha é fundamental para aumentar o conhecimento, promover a autonomia e facilitar o autocuidado, especialmente em um contexto onde as orientações verbais predominam, garantindo que as necessidades educacionais dos usuários de PrEP sejam atendidas de maneira individualizada e centrada na pessoa.

No contexto atual, a luta contra a aids continua há mais de quatro décadas, sendo considerada ainda uma doença repleta de estigmas sociais, especialmente relacionados às minorias sexuais, como as LGBTQIA+^(20)^. Ao longo desse tempo, houve grandes avanços na área da saúde e na área tecnológica, facilitando o acesso a novas informações. De fato, o maior acesso a informações pela população impacta diretamente em questões de saúde pública, uma vez que a alfabetização em saúde proporciona ao indivíduo capacidade de exercer autonomia sobre seus processos de saúde, e fortalecer a tomada de decisões de forma mais consciente e informada^(21)^.

O controle da transmissão do HIV ainda é um grande desafio global e mediante isso, diversas metas foram estabelecidas a fim de tentar atingi-lo. Um exemplo disso, é uma das metas dos ODS, especificamente a meta de saúde e bem-estar, que visa a eliminação da aids até o ano de 2030^(2)^. Dessa forma, um modo de auxiliar a atingir a erradicação da aids é através da alfabetização em saúde da população quanto aos métodos de prevenção. Para que tal processo ocorra, materiais didáticos que facilitem a compreensão das informações se fazem necessários^(22)^.

As tecnologias educativas têm desempenhado um papel fundamental na educação em saúde, especialmente ao direcionar estratégias para a conscientização dos assuntos que tratam de determinados tópicos; através da exposição lúdica e interativa para o público o qual pretende atingir; pode ser redigida com fácil entendimento em mente, utilização de linguagem simplificada e inserção de informações relevantes^(24)^. No caso da cartilha desenvolvida no correr deste estudo, ela exerce a função de conscientizar o público-alvo sobre as lutas de erradicação da aids, prevenção e maior segurança, com a garantia de evidências fidedignas, atualizadas e escritas de maneira acessível, tanto em termos de vocabulário como de fácil interpretação.

Uma cartilha educativa, seja ela no formato impresso ou online, auxilia na dispersão do conhecimento com amplo alcance de indivíduos em ambas as modalidades. A cartilha educativa que tenha como objetivo a alfabetização em saúde - contendo textos de fácil compreensão e imagens autoexplicativas que facilitem o entendimento do material, especialmente - tem o potencial de alcançar populações vulneráveis, uma vez que não necessitam de suporte adjacente, como uma conexão com a internet e aparelhos eletrônicos para leitura. Dessa forma, a cartilha vem sendo uma grande ferramenta para profissionais da saúde, no âmbito de enfrentamento e implementação de estratégias de prevenção em saúde^(9)^.

A cartilha educativa abordou os principais tópicos identificados nas evidências científicas relacionadas à PrEP, como o funcionamento, indicação, eficácia, benefícios e efeitos colaterais, obtendo um Índice de Validade de Conteúdo (S-CVI/UA) Global de 0,94, acima da meta considerada para a aprovação, sendo ela um índice superior a 0,80. Dessa forma, considera-se válida por especialistas da área da saúde e público-alvo para uso futuro como tecnologia educativa com a população, uma vez que, a cartilha foi desenvolvida a fim de disponibilizar informações atualizadas sobre a PrEP de maneira acessível.

A validação com o público-alvo revelou uma avaliação geral positiva da cartilha em relação à escrita, aparência e motivação. Apesar de algumas opiniões sobre as cores, a maioria dos usuários a considerou útil para promover a educação em saúde além do serviço especializado. As cores foram ajustadas para melhor atender ao perfil dos usuários, assim como a escrita dos efeitos colaterais foi simplificada. Essa validação é crucial para identificar lacunas de compreensão e garantir que o material educativo seja acessível e relevante para diferentes níveis de letramento em saúde. Uma apresentação organizada e atrativa do conteúdo é fundamental para uma leitura eficaz, alinhada com os interesses e necessidades dos leitores.

Uma cartilha educativa bem desenvolvida é um recurso valioso em estratégias de educação em saúde, facilitando a compreensão, sendo acessível e reforçando as orientações fornecidas verbalmente pela equipe de saúde. O formato impresso oferece a vantagem de poder ser consultado posteriormente, servindo como apoio para consultas e leituras ao longo do tempo e favorecer a comunicação com a equipe de saúde. Ressalta-se ainda que a cartilha não substitui o profissional de saúde, nas ações de educação em saúde, no entanto, pode facilitar a comunicação e estimular o autocuidado^(24)^.

### Limitações do estudo

A validação da cartilha foi feita com adultos jovens com alto nível educacional já vinculados a um serviço especializado, excluindo grupos com baixa escolaridade. Apesar disso, todas as etapas foram cumpridas. A população validada reflete usuários reais da PrEP no país. Sugere-se criar outras tecnologias educacionais, como jogos e vídeos, para alcançar diferentes grupos populacionais.

### Contribuições para a área

A construção e validação de uma cartilha inédita para usuários da PrEP representa uma importante tecnologia educativa, de baixo custo e de fácil implementação nos serviços de saúde. Pode alcançar uma ampla audiência e ser utilizada em intervenções educativas como estratégia de ensino-aprendizagem pela equipe de enfermagem e outros profissionais de saúde. Este estudo aborda os principais tópicos a serem incluídos nas orientações para o uso da PrEP, destacando a importância do enfermeiro no processo de educação para a saúde.

## CONCLUSÕES

A cartilha educativa sobre a PrEP foi elaborada em cinco tópicos e validada por profissionais experientes e usuários. Sua versão final impressa pode ser utilizada como um recurso complementar às orientações fornecidas por enfermeiros e médicos durante o acompanhamento especializado, auxiliando assim na garantia do acesso universal a serviços de saúde de qualidade. Tal acesso é crucial para o alcance do terceiro dos Objetivos de Desenvolvimento Sustentável da ONU. Além disso, a cartilha visa fortalecer a alfabetização em saúde sobre a PrEP, um método de prevenção do HIV gratuito disponibilizado pelo SUS. Isso capacita os indivíduos a tomarem decisões informadas sobre sua saúde, promovendo, assim, seu empoderamento e autonomia.

## Data Availability

Não se aplica.
